# Manipulating Li_2_S Redox Kinetics and Lithium Dendrites by Core–Shell Catalysts under High Sulfur Loading and Lean‐Electrolyte Conditions

**DOI:** 10.1002/advs.202207442

**Published:** 2023-03-18

**Authors:** Mengmeng Zhen, Kaifeng Li, Mingyang Liu

**Affiliations:** ^1^ State Key Laboratory of Medicinal Chemical Biology Nankai University 300350 Tianjin China; ^2^ School of Energy and Environmental Engineering Hebei University of Technology Tianjin 300071 China

**Keywords:** lean‐electrolyte, Li_2_S deposition/decomposition, lithium dendrites, lithium–sulfur batteries, redox kinetics

## Abstract

For practical lithium–sulfur batteries (LSBs), the high sulfur loading and lean‐electrolyte are necessary conditions to achieve the high energy density. However, such extreme conditions will cause serious battery performance fading, due to the uncontrolled deposition of Li_2_S and lithium dendrite growth. Herein, the tiny Co nanoparticles embedded N‐doped carbon@Co_9_S_8_ core–shell material (CoNC@Co_9_S_8_NC) is designed to address these challenges. The Co_9_S_8_NC‐shell effectively captures lithium polysulfides (LiPSs) and electrolyte, and suppresses the lithium dendrite growth. The CoNC‐core not only improves electronic conductivity, but also promotes Li^+^ diffusion as well as accelerates Li_2_S deposition/decomposition. Consequently, the cell with CoNC@Co_9_S_8_NC modified separator delivers a high specific capacity of 700 mAh g^−1^ with a low‐capacity decay rate of 0.035% per cycle at 1.0 C after 750 cycles under a sulfur loading of 3.2 mg cm^−2^ and a *E*/*S* ratio of 12 µL mg^−1^, and a high initial areal capacity of 9.6 mAh cm^−2^ under a high sulfur loading of 8.8 mg cm^−2^ and a low *E*/*S* ratio of 4.5 µL mg^−1^. Besides, the CoNC@Co_9_S_8_NC exhibits an ultralow overpotential fluctuation of 11 mV at a current density of 0.5 mA cm^–2^ after 1000 h during a continuous Li plating/striping process.

## Graphical abstract

The uncontrolled deposition of Li_2_S and lithium dendrites growth lead to the extremely low sulfur utilization and premature failure of LSBs. Here, this work designs the tiny Co nanoparticles embedded N‐doped carbon@Co_9_S_8_ core–shell catalyst to address these challenges. It as an interlayer material accelerates the Li_2_S deposition–decomposition and alleviates lithium dendrites growth under high sulfur loading and lean‐electrolyte conditions.

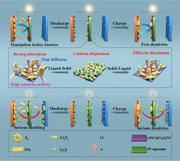

## 1 Introduction

Lithium–sulfur batteries (LSBs) are one of the most promising candidates because of their superior theoretical gravimetric energy densities (2500 Wh kg^−1^), environmental benignity, and abundant sulfur raw materials.^[^ ^1^, ^2^, ^3^, ^4^, ^5^ ^]^ Nevertheless, the severe shuttle effect of lithium polysulfides (LiPSs), poor electronic conductivity of S_8_ and Li_2_S, the sluggish electrochemical reaction kinetics, and uncontrolled lithium dendrite growth lead to the unsatisfactory cycling stability and low sulfur utilization.^[^ ^6^, ^7^, ^8^, ^9^ ^]^ These inherent issues hinder the practical applications of LSBs.^[^ ^10^, ^11^, ^12^, ^13^ ^]^ Numerous polar materials and catalytic materials have been designed as sulfur hosts or interlayer materials to successfully suppress the shuttling and accelerate the conversion of LiPSs.^[^ ^14^, ^15^, ^16^, ^17^, ^18^, ^19^, ^20^ ^]^ It's worth noting that these performance improvements of LSBs are achieved mainly under low sulfur loadings (<3 mg cm^−2^) and excessive electrolyte (high electrolyte/sulfur ratio, *E*/*S* > 15 µL mg_S_^−1^).^[^ ^21^, ^22^, ^23^ ^]^ But the use of high *E*/*S* ratio results in a low practical energy density, greatly weakening the high gravimetric energy density advantages of LSBs.^[^ ^24^, ^25^, ^26^, ^27^, ^28^ ^]^

A low *E*/*S* ratio of less than 5.0 µL mg^−1^ and a high sulfur loading of more than 3.0 mg cm^−2^ are required to achieve the practical energy density of 400 Wh kg^−1^ in LSBs.^[^ ^25^, ^29^ ^]^ However, reducing the *E*/*S* ratio (lean‐electrolyte) inevitably leads to more sluggish electrochemical reaction kinetics, because of the reduction of ionic conductivity in electrolyte with increased viscosity caused by high concentrated LiPSs.^[^ ^30^, ^31^, ^32^ ^]^ Especially under such extreme conditions, the sluggish redox kinetics of Li_2_S causes its uncontrolled deposition and aggravates the interface passivation, inducing extremely low sulfur utilization and premature failure of LSBs.^[^ ^22^, ^33^, ^34^, ^35^ ^]^ Using interlayer between cathode and separator is recognized as an effective strategy to improve the performances of LSBs.^[^ ^1^, ^36^, ^37^ ^]^ Whereas, the wetting of modified separator needs more electrolyte, which requires interlayer materials to have a stronger holding ability for electrolyte, especially under low *E*/*S*. So far, more attentions have been paid to the adsorption and conversion for LiPSs in LSBs under excessive electrolyte. There are few reports on both the Li_2_S deposition/decomposition and electrolyte‐holding ability of interlayer materials in LSBs under lean‐electrolyte and high sulfur loading conditions.

Of note, metal Co and cobalt sulfides (such as CoS_2_, Co_3_S_4_, and Co_9_S_8_) with high surface free energy and abundant active sites, have been shown high catalytic activity to promote the sulfur conversion process in LSBs.^[^ ^6^, ^38^, ^39^, ^40^, ^41^, ^42^ ^]^ For example, Dai and co‐workers^[^ ^43^ ^]^ confirmed that the honeycomb‐like Co_9_S_8_ tubules had high binding energies which could effectively remit the dissolution and promote the redox reaction kinetics of LiPSs. Qian et al.^[^ ^44^ ^]^ introduced a catalytic interlayer, Co nanoparticles embedded in the skeleton surface, improved the Li_2_S conversion, and enhanced the battery performances. Nevertheless, the weak catalytic performance of single Co‐sulfide for Li_2_S_2_/Li_2_S and the poor holding capacity of single Co‐metal for electrolyte affect the performance improvement of LSBs. Additionally, the high sulfur loading causes larger volume expansion and slower Li^+^ diffusion occur during discharge/charge process, which needs interlayer materials to possess more appropriate structure and component. Compared with other structures, the core–shell structure can provide more advantages.^[^ ^45^ ^]^ The hollow shell with higher LiPS adsorption capability would provide enough space to impede the volume expansion and immobilize dissolved LiPSs, and the porous core with stronger catalytic activity would further promote the LiPS transformation process.^[^ ^46^ ^]^ Therefore, how to design a catalytic interlayer material with core–shell structure, which not only effectively captures the LiPSs and hold electrolyte, but also manipulates redox kinetics of Li_2_S, as well as regulate Li dissolution/deposition under lean electrolyte and high sulfur loading conditions, is of great significance for achieving the practical application of LSBs.

Herein, inspired by the properties of Co‐sulfides and Co‐metal, a core–shell material, tiny Co nanoparticles embedded within N‐doped carbon (CoNC) as core and Co_9_S_8_ nanoparticles embedded within N‐doped carbon (Co_9_S_8_NC) as shell (noted as CoNC@Co_9_S_8_NC), is designed and constructed. It is introduced as a promising catalyst for improving the redox kinetics of Li_2_S in LSBs. Experimental results and density functional theory (DFT) calculation analysis reveal that (1) the Co_9_S_8_NC‐shell possesses high binding energy for electrolyte molecule and LiPSs which can effectively capture LiPSs and hold electrolyte, (2) the CoNC‐core not only improves electronic conductivity, but also promotes Li^+^ diffusion as well as accelerates Li_2_S deposition/decomposition, (3) the CoNC@Co_9_S_8_NC material can efficiently control homogeneous distribution of Li^+^ flux to suppress the growth of Li dendrite, and (4) the core–shell structure can provide buffer space for volume expansion and spatial constraint to the LiPSs. Consequently, the CoNC@Co_9_S_8_NC as catalytic interlayer delivers a high specific capacity of ≈700 mAh g^−1^ at a current density of 1.0 C after 750 cycles under a high sulfur loading of ≈3.2 mg cm^−2^ and a low *E*/*S* of 12 µL mg^−1^. Besides, it shows a high initial areal capacity of 9.6 mAh cm^−2^ under the ultralow *E*/*S* ratio of 4.5 µL mg^−1^ and high sulfur loading of 8.8 mg cm^−2^. The CoNC@Co_9_S_8_NC can also stabilize the Li plating/striping on Li anodes during a continuous Li plating/striping process.

## 2 Results and Discussion

### 2.1 Preparation and Characterization of the CoNC@Co_9_S_8_NC

**Figure 1:**
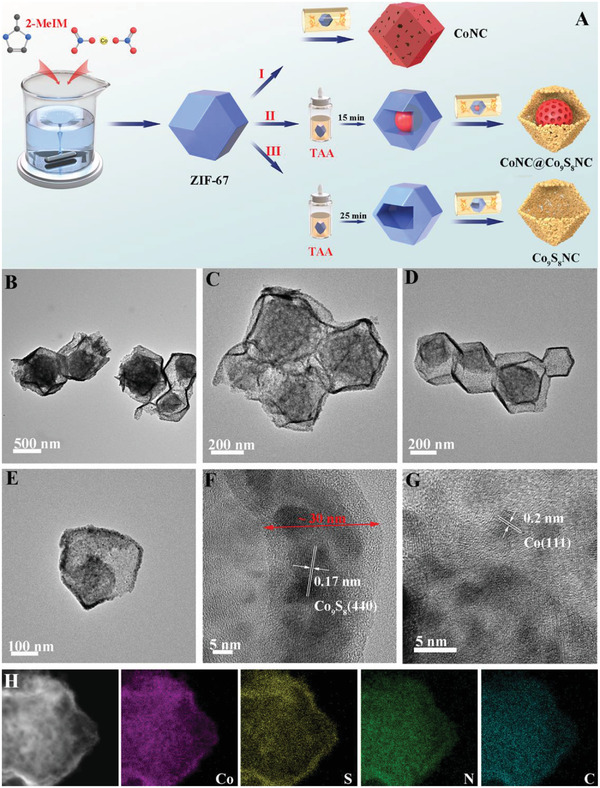
A) Schematic illustration for the CoNC, CoNC@Co_9_S_8_NC, and Co_9_S_8_NC. B–E) Transmission electron microscopy (TEM) images of CoNC@Co_9_S_8_NC. F,G) High‐resolution TEM (HRTEM) images of CoNC@Co_9_S_8_NC. H) Elemental mappings of CoNC@Co_9_S_8_NC.

The construction process of the CoNC@Co_9_S_8_NC material is presented in **Figure** **1**A. ZIF‐67 polyhedrons were initially prepared by the self‐assembly of 2‐methylimidazole (2‐MeIM) and cobalt nitrate in methanol. The ultrafine Co nanoparticles embedded within N‐doped carbon (noted as CoNC) could be obtained through directly annealing ZIF‐67 polyhedrons under Ar atmosphere flow (Step I). If the ZIF‐67 polyhedrons first reacted with thioacetamide (TAA) for 15 min in ethanol and then annealed under Ar atmosphere, it would get the CoNC@Co_9_S_8_NC core–shell polyhedrons (Step II). During the reaction process between external ZIF‐67 and TAA, amorphous Co‐sulfide shells were formed and coated around the internal residual ZIF‐67 cores.^[^ ^47^ ^]^ After annealing under Ar atmosphere flow, the amorphous Co‐sulfide shells and internal residual ZIF‐67 cores were converted to the Co_9_S_8_NC and CoNC, respectively. By increasing the reaction time with TAA, the entire ZIF‐67 from outside to inside was vulcanized and then was calcined to obtain hollow Co_9_S_8_NC material (Step III).

Scanning electron microscopy (SEM) and transmission electron microscopy (TEM) images show that ZIF‐67 possesses a uniform polyhedral morphology with an average size of 300–400 nm (Figure S1A,B, Supporting Information). Figure S1C (Supporting Information) exhibits a typical powder X‐ray diffraction (XRD) pattern, which can be indexed to the ZIF‐67.^[^ ^48^ ^]^ From SEM images, it can be observed that the smooth surface of ZIF‐67 becomes rough (Figure S2A, Supporting Information). As shown in TEM images (Figure S2B,C, Supporting Information) and the XRD pattern (Figure S2D, Supporting Information), the polyhedral ZIF‐67 turns to be many ultrafine Co nanoparticles embedded within N‐doped carbon skeleton (CoNC) and the phase converts to Co‐metal phase after annealing. The core–shell structures of CoNC@Co_9_S_8_NC can be also clearly seen in the SEM and TEM images. The cracked CoNC@Co_9_S_8_NC polyhedrons reveal obvious core–shell structures and rough surface in Figure S3B–D (Supporting Information). The TEM images (Figure 1B–D) present the core–shell structural feature with the outer shell thickness of ≈30 nm and the inner core size of ≈200 nm, respectively. High‐resolution TEM (HRTEM) images (Figure 1E,F) further show that many nanoparticles of ≈10 nm make up the outer shell of Co_9_S_8_NC. The lattice fringes of the outer shell and inner core of CoNC@Co_9_S_8_NC (Figure 1F,G) are measured to be 0.17 and 0.2 nm, which can be well indexed to Co_9_S_8_ (440) and Co (111) planes, respectively.^[^ ^40^, ^49^ ^]^ The elemental mappings (Figure 1H) of Co, S, N, and C further reveal the distribution of homogeneous elements in the CoNC@Co_9_S_8_NC. With the increase of vulcanizing time, the inner core CoNC also transformed into the outer shell Co_9_S_8_NC, and then formed the hollow Co_9_S_8_NC polyhedrons, as revealed in Figure S4 (Supporting Information).

**Figure 2:**
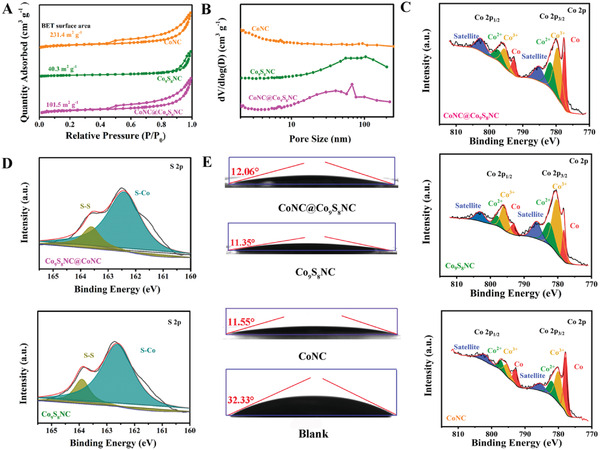
A) N_2_ absorption/desorption isotherm, B) pore size distributions, C) high‐resolution Co 2p, and D) S 2p X‐ray photoelectron spectroscopy (XPS) spectra of different interlayer materials and contact angles between electrolyte and different modified separator.

**Figure** **2**A,B shows the N_2_ absorption–desorption isotherm and pore size distributions of the three interlayer materials. The Brunauer–Emmett–Teller (BET) surface area of CoNC (231.4 m^2^ g^−1^) and CoNC@Co_9_S_8_NC (101.5 m^2^ g^−1^) is higher than that of Co_9_S_8_NC (40.3 m^2^ g^−1^), which is attributed to partial agglomeration of Co_9_S_8_ after vulcanization and recrystallization, and the dispersed state of CoNC. The average diameters of Co_9_S_8_NC, CoNC@Co_9_S_8_NC and CoNC were calculated to be 21.9, 13.5 nm, and 5.2 nm, respectively, revealing the presence of abundant pores. X‐ray photoelectron spectroscopy (XPS) spectrum (Figure S5A, Supporting Information) reveals five peaks in CoNC@Co_9_S_8_NC and Co_9_S_8_NC, which are assigned to C 1s, O 1s, N1s, Co 2p, and S 2p, respectively. The high‐resolution N 1s XPS spectra (Figure S5B, Supporting Information) of CoNC, CoNC@Co_9_S_8_NC and Co_9_S_8_NC reveal the three peaks at 398.5, 399.9, and 400.8 eV, corresponding to pyridinic N, pyrrolic N, and graphitic N, respectively.^[^ ^50^ ^]^ As shown in Co 2p XPS spectrum (Figure 2C), the prominent peaks could be deconvoluted into Co 2p_1/2_ and Co 2p_3/2_, where the peaks at 798.3 and 782.2 eV are ascribed to Co^2+^, the peaks at 794.7 and 779.5 eV are matched to Co^3+^, while the peaks at 793.1 and 778.7 eV are attributed to Co^0^, separately.^[^ ^51^ ^]^ The reduction of peak intensity for Co^0^ and the increased of peak intensity for Co ^2+^ and Co^3+^ in the high‐resolution Co 2p XPS spectrum of CoNC, CoNC@Co_9_S_8_NC and Co_9_S_8_NC, indicate the formation of outer shells Co_9_S_8_ in CoNC@Co_9_S_8_ and the disappear of inner core Co in Co_9_S_8_. The peaks at 162.5 and 163.8 eV in high‐resolution S 2p XPS spectra (Figure 2D) show the Co‐S bond and S‐S bond in CoNC@Co_9_S_8_NC and Co_9_S_8_NC. The high relative intensity of peaks located at ≈1320 cm^−1^ (D‐band) and ≈1590 cm^−1^ (G‐band) (*I*_D_/*I*_G_) in Raman spectra (Figure S6, Supporting Information) suggests the high graphitization degree for CoNC, CoNC@Co_9_S_8_NC, and Co_9_S_8_NC.

To enhance the intercept ability of conventional polypropylene (PP) separator for LiPSs, CoNC@Co_9_S_8_NC as interlayer materials was coated on the surface of PP separator. As shown in SEM images (Figure S7A,B, Supporting Information), the surface of blank separator has many pores, and these pores are covered after coating with CoNC@Co_9_S_8_NC. From the cross‐section for CoNC@Co_9_S_8_NC modified separator, the coating thickness of the interlayer materials is ≈10 µm (Figure S7C, Supporting Information). Besides, no obvious cracks are found in the CoNC@Co_9_S_8_NC modified separator after folding (Figure S7D, Supporting Information). From the Figure S7E,F (Supporting Information), CoNC@Co_9_S_8_NC materials are fully covered on the surface of the PP separator. There are no obvious pores on the surface of CoNC@Co_9_S_8_NC modified separator after coating. To assess the wettability of electrolyte on the modified separator, the contact angles were tested. Compared with the contact angle between electrolyte and blank separator (32.33°), that between electrolyte and CoNC@Co_9_S_8_NC (12.06°), CoNC (11.55°), and Co_9_S_8_NC (11.35°) modified separator is much smaller, suggest that these interlayer materials can enhance electrolyte wettability and ensure LiPSs utilization under high sulfur loading and lean electrolyte conditions.

### 2.2 Electrochemical Performances of the CoNC@Co_9_S_8_NC as Interlayer Materials for LSBs

**Figure 3:**
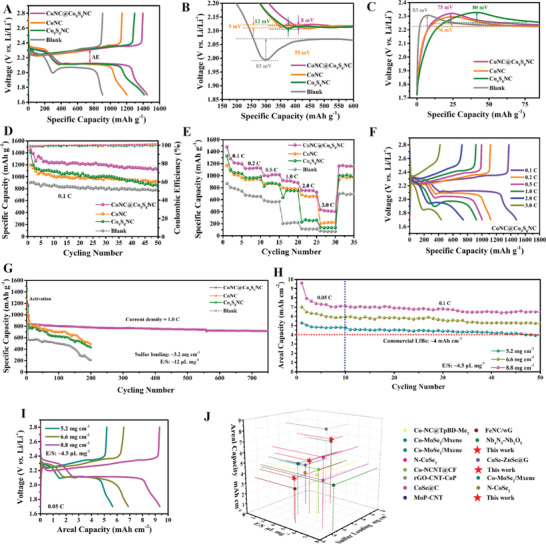
A–C) Charge–discharge curves, D) cycling performances, E) rate capabilities, and F) charge–discharge curves at different current rates, and G) long‐term cycling performances of the cells with different modified separators under a sulfur loading of 3.2 mg cm^−2^ and *E*/*S* ratios of 12 µL mg^−1^. H) Cycling stability and I) charge–discharge curves of the cell with CoNC@Co_9_S_8_NC modified separator via areal capacities at *E*/*S* ratios of 4.5 µL mg^−1^. J) Comparison of the sulfur loading, areal capacity, and *E*/*S* ratio among this work and recently reported similar materials.

Electrochemical performances of LSBs with S cathode (sulfur loading = 3.2 mg cm^−2^) and different modified separators under *E*/*S* = 12 µL mg^−1^ were investigated to evaluate the effect of CoNC@Co_9_S_8_NC. The charge–discharge profiles (**Figure** **3**A) of the cells with CoNC@Co_9_S_8_NC, Co_9_S_8_NC, CoNC, and blank modified separators show two obvious discharge plateaus at a current density of 0.1 C. Compared with the cells with Co_9_S_8_NC (1296/1412 mAh g^−1^, 158 mV), CoNC (1143/1198 mAh g^−1^, 153 mV), and blank (904/905 mAh g^−1^, 228 mV) modified separators, the cell with CoNC@Co_9_S_8_NC modified separator exhibits the smallest polarization potential (Δ*E*, 151 mV) and the highest initial charge–discharge specific capacities of 1391/1448 mAh g^−1^. The valley between the first and second discharge plateau is considered as the Li_2_S nucleation point. The cell with CoNC@Co_9_S_8_NC modified separator shows the smallest overpotentials of 8 mV among that with Co_9_S_8_NC (12 mV), CoNC (9 mV), and blank (83 mV) interlayers (Figure 3B). The oxidation of the insulating Li_2_S generally requires particular overpotential to react. As presented in Figure 3C, the onset potential of the cell with CoNC@Co_9_S_8_NC (75 mV) modified separator also is the lowest as compared to that with CoNC (76 mV), Co_9_S_8_NC (80 mV), and blank (83 mV) modified separator. These results indicate that CoNC@Co_9_S_8_ not only can effectively accelerate the solid−solid conversion and the nucleation of Li_2_S, but also can promote the solid−liquid conversion and the dissolution of Li_2_S. Additionally, its discharge specific capacity keeps at 1120 mAh g^−1^ and a coulombic efficiency with ≈100% at a current density of 0.1 C after 50 cycles, which are better than that with Co_9_S_8_ (845 mAh g^−1^), CoNC (926 mAh g^−1^) and blank (774 mAh g^−1^) modified separators (Figure 3D). It can be seen from Figure 3E that the cell with CoNC@Co_9_S_8_NC modified separator presents the best rate capability with the highest average specific capacities of 1275, 1124, 1009, 904, 749 and 416, mAh g^−1^ at 0.1, 0.2, 0.5, 1.0, 2.0, and 3.0 C, respectively. When the current rate recovers to 0.1 C, its discharge capacity still owns 1160 mAh g^−1^. Furthermore, this cell shows evident plateaus in discharge curves (Figure 3F) and low Δ*E* (Figure S8D, Supporting Information) at different current rates, even at 3.0 C, indicating the good structural stability and catalytic activity of CoNC@Co_9_S_8_NC. By contrast, other cells with Co_9_S_8_NC, CoNC, and blank modified separators present relatively high Δ*E* and poor rate performances at 0.1, 0.2, 0.5, 1.0, 2.0, and 3.0 C in Figure S8 (Supporting Information), respectively. The mass ratios of CoNC and Co_9_S_8_NC in the CoNC@Co_9_S_8_NC were ≈2:7 by using inductively coupled plasma optical emission spectrometry (ICP) tests. We physically mixed CoNC and Co_9_S_8_NC with the mass ratio to obtain CoNC‐Co_9_S_8_NC. To investigate the structural advantages of the core–shell CoNC@Co_9_S_8_NC, the cell with CoNC‐Co_9_S_8_NC modified separator shows lower initial charge–discharge specific capacities of 1011/1132 mAh g^−1^, larger Δ*E* (163 mV) and the onset potentials (15 and 82 mV) at 0.1 C than these with CoNC, Co_9_S_8_NC, and CoNC@Co_9_S_8_NC modified separators, as shown Figure S9A–C (Supporting Information). Compared with CoNC@Co_9_S_8_NC, CoNC‐Co_9_S_8_NC also presents a poor cycling performance with specific capacity of 889 mAh g^−1^ at 0.1 C after 50 cycles (Figure S9D, Supporting Information), and inferior rate capacities with low ΔE from 0.1 to 3.0 C (Figure S9E,F, Supporting Information). The phenomenon indicates that the mixture of CoNC and Co_9_S_8_NC cannot realize the synergistic effect of composition and structure.

The long‐term performances of these cells with different modified separators under a high sulfur loading of 3.2 mg cm^−2^ and a *E/S* ratio of 12 µL mg^−1^ were investigated at a current density of 1.0 C. As shown in Figure 3G, the cell with CoNC@Co_9_S_8_NC modified separator after activation delivers a high discharge specific capacity of 953 mAh g^−1^, and maintains ≈700 mAh g^−1^ in the 750th cycle with a low‐capacity decay of 0.035% per cycle at 1.0 C. By contrast, the cells with Co_9_S_8_NC (430 mAh g^−1^), CoNC (486 mAh g^−1^), and blank (210 mAh g^−1^) modified separators exhibit poor cyclic stability and low specific capacities after 200 cycles. Additionally, the cycling properties of LSBs under high sulfur loading and ultralow *E*/*S* ratios conditions are an important evaluation factors for achieving high energy density. The cell with CoNC@Co_9_S_8_NC modified separator under the sulfur loading of 5.6, 6.6, and 8.8 mg cm^−2^, and an ultralow *E*/*S* ratio of 4.5 µL mg^−1^ at 0.05 C delivers high initial areal capacities with 5.3, 7.0, and 9.6 mAh cm^−2^, respectively. After activated for 10 cycles at 0.05 C, the cell retains a high areal capacity of 4.0 mAh cm^−2^ after 50 cycles under the sulfur loading of 5.2 mg cm^−2^ and an *E*/*S* of 4.5 µL mg^−1^ at 0.1 C. Moreover, increasing sulfur loading to 6.6 and 8.8 mg cm^−2^, it still delivers a high areal capacity of 5.3 and 6.5 mAh cm^−2^ at 0.1 C after 50 cycles (Figure 3H), respectively. It with high sulfur loadings still presents obvious two‐plateau voltage profiles under an *E*/*S* of 4.5 µL mg^−1^, as shown in Figure 3I. These superior cycling performances with CoNC@Co_9_S_8_NC interlayer under high sulfur loading and lean‐electrolyte conditions is a considerable value as compared to other similar materials (Figure 3J).^[^ ^6^, ^21^, ^38^, ^52^, ^53^, ^54^, ^55^, ^56^, ^57^, ^58^, ^59^ ^]^

### 2.3 LiPSs Adsorption Characterization

**Figure 4:**
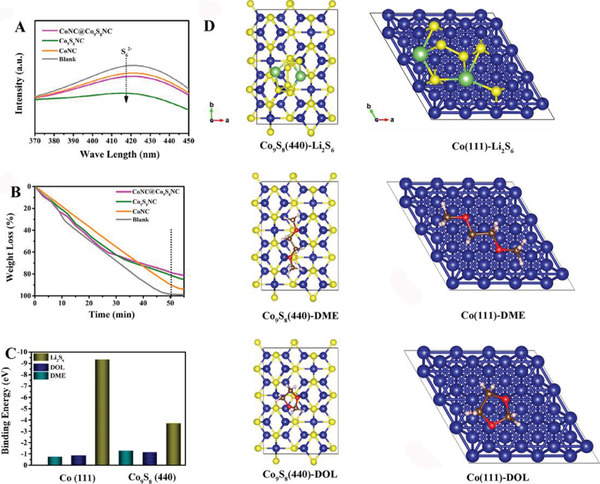
A) UV–Vis spectra of Li_2_S_6_ (10 M) treated with blank, CoNC@Co_9_S_8_NC, Co_9_S_8_NC, and CoNC materials. B) The weight loss tests of electrolyte (DOL/DME = 1:1) immersed in blank, CoNC@Co_9_S_8_NC, Co_9_S_8_NC, and CoNC materials at room temperature. C) Binding energies between Li_2_S_6_/DME/DOL and Co(111)/Co_9_S_8_(440). D) Optimized structures of Li_2_S_6_/DME/DOL and Co(111)/Co_9_S_8_(440). The C, O, H, Co, and S atoms were represented as brow, red, pink, blue, and yellow, respectively.

Based on the superior battery performances, the corresponding mechanisms of the CoNC@Co_9_S_8_NC interlayer are deeply analyzed. The absorption ability of different interlayers for LiPSs was evaluated by the adsorption test and UV–Vis spectra analysis. From digital photos (**Figure** **4**A and Figure S10A, Supporting Information), the color of Li_2_S_6_ solution treated with CoNC@Co_9_S_8_NC and Co_9_S_8_NC became lighter than that treated with CoNC after 6 h. The UV–Vis spectra of Li_2_S_6_ solution treated with CoNC@Co_9_S_8_ and Co_9_S_8_NC also show lower peak intensity among these interlayer materials. As shown in the digital photos, the surface of CoNC@Co_9_S_8_ modified separators after cycling also displays the smallest area of LiPSs among these separators (Figure S10B, Supporting Information). The practical interception ability of soluble Li_2_S_6_ solution through the PP separators coated with different interlayer materials was assessed by an H‐shaped permeating. As shown in Figure S11 (Supporting Information), the transparent solutions on the right side with blank PP separators turned yellow after 6 h permeation. In this respect, very little Li_2_S_6_ solution crossed the CoNC@Co_9_S_8_NC and Co_9_S_8_NC modified separators to the right side after 24 h, indicative of the effective inhibition of CoNC@Co_9_S_8_NC and Co_9_S_8_NC materials for the random diffusion of soluble LiPSs. These results suggest that CoNC@Co_9_S_8_NC and Co_9_S_8_NC have stronger adsorption ability for LiPSs compared with CoNC.

The electrolyte‐holding ability of interlayer materials in lean‐electrolyte LSBs especially ether‐based electrolyte is an important evaluation index. The weight loss for the mixture of the interlayer materials and electrolyte (DOL/DME = 1:1) is shown in Figure 4B. The electrolyte without adding materials was evaporated completely within 51 min. By contrast, the weight loss ratios of electrolyte upon addition of CoNC@Co_9_S_8_NC, Co_9_S_8_NC and CoNC after 51 min were 79.3%, 81.9%, and 89.7%, respectively. Besides, DFT calculations were performed to further investigate the LiPSs adsorbability and electrolyte‐holding ability of these interlayer materials. Figure 4D shows the optimized structures of the Li_2_S_6_/DOL/DME bound system with the surface of Co(111)/Co_9_S_8_(440) and the corresponding binding energies. As presented in Figure 4C, the binding energies between DOL/DME and the surface of Co_9_S_8_(440) (−1.15 eV/−1.27 eV) are found to be higher than that between DOL/DME and the surface of Co(111) (−0.87 eV/−0.75 eV). However, compared with the surface of Co_9_S_8_(440) (−3.69 eV), the surface of Co(111) shows too much higher binding energy for Li_2_S_6_ (−9.35 eV). According to the optimized structural model, the Li_2_S_6_ with a complete molecular structure can be adsorbed on the surface of Co_9_S_8_(440). While Li_2_S_6_ will spontaneously dissociate on the surface of Co(111), thus significantly increasing its binding energy. Too binding energy for Li_2_S_6_ will hinder the next electrochemical reaction. These results indicate that CoNC@Co_9_S_8_NC and Co_9_S_8_NC have stronger binding interaction with electrolyte molecules and more suitable adsorption effect for LiPSs than CoNC, and can effectively capture LiPSs and enhance the electrolyte‐holding ability under lean electrolytes.

### 2.4 Li^+^ Diffusion Characterization

**Figure 5:**
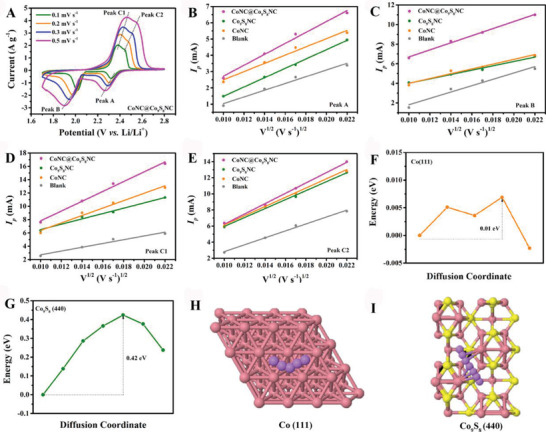
A) Cyclic voltammetry (CV) curves of the cell with CoNC@Co_9_S_8_NC modified separators at scanning rates from 0.1 to mV s^−1^. B–E) Plots of peak currents (*I*_p_) with the square root of the scan rate (*ν* ^1/2^) for different CV peaks during discharge–charge process. F–I) Energy profiles and minimum energy path for Li^+^ diffusion on the surface of Co (111) and Co_9_S_8_ (440). The purple, yellow, and pink balls represent Li, S and Co atoms, respectively.

It is well known that introducing electrocatalyst into LSBs can promote LiPSs redox kinetics. To understand the catalytic effect of interlayer materials, a series of systematic electrochemical measurements was performed. Cyclic voltammetry (CV) curves of the cells with different interlayers and same sulfur loading (≈3.2 mg cm^−2^) at a scanning rate of 0.1 mV s^–1^ are presented in Figure S12A (Supporting Information). The CV curves for the cell with CoNC@Co_9_S_8_NC modified separators exhibit two anodic peaks at 2.35 and 2.43 V and two cathodic peaks at 2.32 and 2.01 V, respectively. These CV peaks are ascribed to the corresponding conversions of Li_2_S_2_/Li_2_S to LiPSs, LiPSs to S_8_, S_8_ to LiPSs, and LiPSs to Li_2_S_2_/Li_2_S, respectively.^[^ ^60^ ^]^ The sharpest redox peaks and the smallest polarization for the cell with CoNC@Co_9_S_8_NC modified separators indicate the enhanced redox reaction kinetics of multiple conversion reaction. Lithium‐ion (Li^+^) diffusion property is one of important factors affecting sulfur redox kinetics.^[^ ^61^ ^]^ It can be qualitatively determined by CV tests of these cells with CoNC@Co_9_S_8_NC, CoNC, and Co_9_S_8_NC modified separators at various scanning rates from 0.1 to 0.5 mV s^–1^, as shown in **Figure** **5**A and Figure S12B–D (Supporting Information). The Li^+^ diffusion properties can be described by the Randles–Sevcik equation.^[^ ^62^ ^]^

(1) $$I_{p}=2.69\times 10^{5}n^{1.5}{\mathrm{AD}}_{\mathrm{Li}+}^{0.5}v^{0.5}C$$

It illustrates that the corresponding Li^+^ diffusion coefficient (*D*_Li_) can be estimated from the proportional relationship between the slope of the *I*_p_ (peak current) and the square root of the *v* (scanning rate). The fitted lines for the *I*_p_ versus *v^0.5^* are shown in Figure 5B–E. It can be observed that the cell with CoNC@Co_9_S_8_NC modified separator possesses the sharpest slopes among these cells, meaning the largest *D*_Li_ for CoNC@Co_9_S_8_NC. Besides, The Li^+^ diffusion mechanism at CoNC@Co_9_S_8_NC modified separator was further studied by DFT calculations of the Li^+^ diffusion barrier energies on the surface of Co_9_S_8_ (440) and Co (111). The minimum energy paths and the corresponding geometrical configurations of Li^+^ on the surface of Co_9_S_8_ (440) and Co (111) are shown in Figure 5H,I. The diffusion barrier of Li^+^ on the surface of Co (111) is only 0.01 eV (Figure 5F), which is much 40 times lower than that on the surface of Co_9_S_8_ (440) (0.42 eV, Figure 5G). These experimental results and DFT calculations reveal that CoNC@Co_9_S_8_NC and CoNC as electrocatalysts are beneficial to the accelerate Li^+^ diffusion, thus facilitates redox kinetics in LSBs.

### 2.5 Catalytic Conversion and Lithium Dendrite Characterization

**Figure 6:**
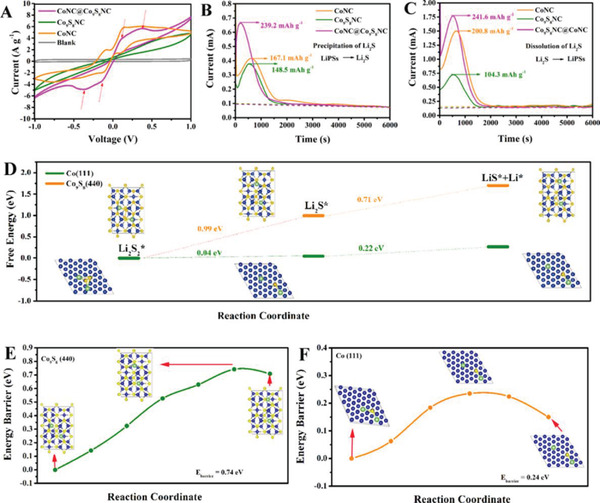
A) Cyclic voltammetry (CV) curves of the symmetric cells with different interlayer materials at a scanning rate of 0.5 mV s^−1^. B,C) Deposition and dissolution kinetics of Li_2_S on different interlayer materials. D) Energy profiles for the reduction of Li_2_S_2_ and decomposition of Li_2_S cluster on the surfaces of Co (111) and Co_9_S_8_ (440) with the optimized adsorption configuration. E,F) Decomposition processes of Li_2_S cluster on the surfaces of Co (111) and Co_9_S_8_ (440) with the initial, transition, and final structures. The blue, yellow, and green balls represent Co, S, and Li atoms, respectively.

Symmetrical cells with CoNC@Co_9_S_8_NC/Co_9_S_8_NC/CoNC electrodes and Li_2_S_6_‐contained electrolyte were also assembled and tested by CV. The CV curve (**Figure** **6**A) of CoNC@Co_9_S_8_NC at a scanning rate of 0.5 mV s^−1^ shows two pairs of distinct redox peaks, attributing to the two‐steps reduction reaction of Li_2_S_6_ to Li_2_S and the oxidation reaction of Li_2_S_6_ to S_8_, indicating the superior electrocatalytic capability of CoNC@Co_9_S_8_NC for redox reaction of Li_2_S.^[^ ^63^ ^]^ Among these symmetrical cells, CoNC@Co_9_S_8_NC displays the smallest polarization and the strongest redox peaks, which suggest that CoNC@Co_9_S_8_NC can effectively promote the conversion of LiPSs → Li_2_S. Besides, the redox peaks of CoNC@Co_9_S_8_NC at a large scanning rate of 20 mV s^−1^ also show higher response in comparison with CoNC and Co_9_S_8_NC, as shown in Figure S13 (Supporting Information). This further verifies the good electrochemical kinetics of CoNC@Co_9_S_8_NC.

The Li_2_S deposition and dissolution reactions were analyzed by potentiostatic discharge and charge profiles. As shown in Li_2_S nucleation (Figure 6B and dissolution curves (Figure 6C), the redox peaks appear the earliest and their intensity is the highest for the cell with CoNC@Co_9_S_8_NC among these cells. It also delivers larger Li_2_S‐precipitaion/dissolution capacities of 239.2/241.6 mAh g_s_^−1^ as compared to those with CoNC (167.1/200.8 mAh g_s_^−1^) and Co_9_S_8_NC (148.5/104.3 mAh g_s_^−1^). The results suggest that CoNC@Co_9_S_8_NC has an optimal electrocatalytic activity and more sufficient nucleation and dissolution of Li_2_S. DFT calculations were carried out to better understand the redox reaction mechanism of Li_2_S. The conversion reaction of Li_2_S_2_ to Li_2_S (solid to solid) is considered as the rate‐limiting step for the overall discharge reaction. Optimized structures of the sulfur species on surfaces of Co_9_S_8_ (440) and Co (111) and the values of free energies are revealed in Figure 6D. The free energy of the reduction reaction of Li_2_S_2_ to Li_2_S on the Co (111) surface is only 0.04 eV, that is markedly lower than that on the Co_9_S_8_ (440) surface (0.99 eV). This indicates that the Co (111) is more conducive to promote the conversion of Li_2_S_2_ to Li_2_S as compared to Co_9_S_8_ (440). The free energy required for Li_2_S decomposition on Co_9_S_8_ (440) surface (0.71 eV) during reverse oxidation reaction is much higher than that on Co (111) surface (0.22 eV). Additionally, the energy barriers of Li_2_S dissociation into LiS* and Li* on the surfaces of Co (111) (Figure 6E) and Co_9_S_8_ (440) (Figure 6F) are calculated to be 0.24 and 0.74 eV, respectively. Therefore, the Li_2_S deposition and dissolution curves and DFT calculations reveal that the deposition of Li_2_S and the dissociation of the Li–S bond on the surfaces of CoNC@Co_9_S_8_NC and CoNC are more favorable. The electrochemical impedance spectroscopy (EIS) was constructed for assessing the difference of electronic conductivity for the four interlayers, as presented in Figure S14 (Supporting Information). In the Nyquist plots, the cell with CoNC@Co_9_S_8_NC interlayer in the high‐frequency shows smaller charge–transfer resistance (*R*_ct_) compared with the other cells. Both Co_9_S_8_NC and CoNC have high electrical conductivity due to the formation of NC skeleton in the two materials. By constrast, the Co has a lower electrical resistivity than Co_9_S_8_.^[^ ^63^ ^]^ Moreover, the porous core–shell structure of CoNC@Co_9_S_8_NC is more conducive to electron transport and electrolyte permeation than the solid structure of CoNC. The combination of composition and structural advantages gives the CoNC@Co_9_S_8_NC stronger electronic conductivity and better interfacial kinetics than Co_9_S_8_NC and CoNC.

**Figure 7:**
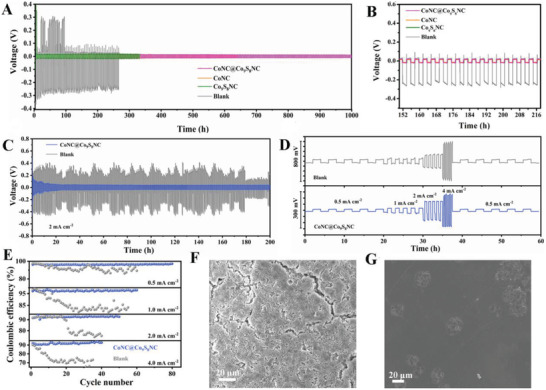
A) Voltage–time curves of Li//Li symmetric cells with different interlayers at 0.5 mA cm^–2^. B) The selected voltage profiles for 152th–216th. C) Voltage–time curves of Li//Li symmetric cells with CoNC@Co_9_S_8_NC interlayer and blank at 2.0 mA cm^–2^ and D) various current densities (0.5–4.0 mA cm^–2^). E) The Coulombic efficiency of the cell with CoNC@Co_9_S_8_NC interlayer and blank at various current densities (0.5–4.0 mA cm^–2^). Scanning electron microscopy (SEM) images of Li anode in the cell with blank (F) and CoNC@Co_9_S_8_NC interlayer (G) after cycling.

The voltage profiles of symmetric cells (Li//Li) with different modified separators were measured at a current density of 0.5 mA cm^–2^ to evaluate the role of the CoNC@Co_9_S_8_NC interlayer on regulating the Li plating/stripping. From the **Figure** **7**A,B, it can be seen that the symmetric cell with blank interlayer shows large polarization overpotential (>200 mV) with fluctuations and short‐circuits after 210 h. Among these separators, the symmetric cell with the CoNC@Co_9_S_8_NC modified separator delivers the smallest overpotential of Li stripping/plating (11 mV) after 1000 h. At 4.0 mA cm^–2^, the cell with CoNC@Co_9_S_8_NC interlayer continuously delivers a lower and more stable overpotential over 200 h than that with blank interlayer (Figure 7C). Rate performances of the symmetric cells with different interlayers were also tested at various current densities from 0.5 to 4.0 mA cm^–2^, as shown in Figure 7D. The cell with CoNC@Co_9_S_8_NC interlayer reveal lower overpotentials of 8.2, 15.8, 76.6, and 122.3 mV at 0.5, 1.0, 2.0, and 4.0 mA  cm^–2^ than that with blank interlayer (34.9, 522.2, 140.2, and 367.7 mV), respectively. When the current density recovers to 0.5 mA cm^–2^, its overpotential still could be boosted to 10.5 mV. Oppositely, the cell with blank interlayer shows higher overpotential and larger fluctuant at 4.0 mA cm^–2^. Moreover, the cell with CoNC@Co_9_S_8_NC interlayer at various current densities from 0.5 to 4.0 mA cm^–2^ also show higher Coulombic efficiencies as comparison to that with blank interlayer (Figure 7E). Especially at 4.0 mA cm^–2^, it still maintains a high Coulombic efficiency of ≈95% after 50 cycles. To further investigate the surface morphology of the Li anodes, the symmetric cells with different modified separators after cycling were disassembled. From SEM images, it could be clearly observed that the Li anode surface of symmetric cell with blank separators is loose and rough with the obvious Li dendrite formation (Figure 7F). In comparison, the Li anode surface of symmetric cell with CoNC@Co_9_S_8_NC interlayer presents smooth surface with a little Li dendrite formation (Figure 7G) after cycling of 200 h. These results indicate that the CoNC@Co_9_S_8_NC materials are beneficial to control the homogeneous distribution of Li^+^ flux and then stabilize the process of Li striping/plating.

## 3 Conclusion

Benefiting from the special structure and ideal composition of CoNC@Co_9_S_8_NC, the Co_9_S_8_NC‐shell with strong absorbability can effectively capture LiPSs and hold electrolyte, and the CoNC‐core can promote Li^+^ diffusion and accelerates Li_2_S redox kinetics. Besides, it also can efficiently suppress the growth of lithium dendrite. Consequently, the cell with CoNC@Co_9_S_8_NC interlayer deliver a superior long‐term cycling performance with a high specific capacity of ≈700 mAh g^−1^ with a low‐capacity decay rate of 0.035% per cycle at 1.0 C after 750 cycles under a high sulfur loading of 3.2 mg cm^−2^ and a *E*/*S* ratio of 12 µL mg^−1^. More importantly, it still reaches to 6.5 mAh cm^–2^ even at a high sulfur areal loading of 8.8 mg cm^–2^ and an ultralow *E*/*S* ratio of 4.5 µL mg^–1^ after 50 cycles. Li//Li cells with the CoNC@Co_9_S_8_NC interlayer also exhibit a good stability and an ultralow over‐potential fluctuation of 11 mV at a current density of 0.5 mA cm^–2^ after 1000 h during a continuous Li plating/striping process. This work provides a good strategy in manipulating Li_2_S redox kinetics and lithium dendrites for LSBs under high sulfur loading and lean‐electrolyte conditions.

## 4 Experimental Section

The preparation process of these materials is based on the improvement.^[^ ^47^ ^]^

### Preparation of ZIF‐67 polyhedrons

Ana amount of 4 × 10^−3^ m Co(NO_3_)_2_·6H_2_O was dissolved in 50 mL methanol and 16 × 10^−3^ m 2‐methylimidazole was dissolved in 50 mL methanol, respectively. Then, the as‐formed two solutions were rapidly mixed and magnetic stirred for 24 h at 35 °C. The precipitate was washed by ethanol for several times and dried at 60 °C for 4 h to obtain ZIF‐67.

### Preparation of CoNC

The CoNC was obtained through directly annealing ZIF‐67 polyhedrons under Ar atmosphere flow for 2 h at 600 °C with a ramp rate of 5 °C min^−1^.

### Preparation of CoNC@Co_9_S_8_NC

An amount of 60 mg ZIF‐67 polyhedrons and 64 mg TAA were dissolved in 40 mL ethanol for 15 min at 85 °C under sealed condition to obtain the precursor‐CoNC@Co_9_S_8_NC. The CoNC@Co_9_S_8_NC was prepared by annealing precursor under Ar atmosphere flow for 2 h at 600 °C with a ramp rate of 5 °C min^−1^.

### Preparation of Co_9_S_8_NC

An amount of 60 mg ZIF‐67 polyhedrons and 64 mg TAA were dissolved in 40 mL ethanol for 25 min at 85 °C under sealed condition to obtain the precursor‐*@*Co_9_S_8_NC. The Co_9_S_8_NC was prepared by annealing precursor under Ar atmosphere flow for 2 h at 600 °C with a ramp rate of 5 °C min^−1^.

## Conflict of Interest

The authors declare no conflict of interest.

## Supporting information

Supporting Information

## Data Availability

The data that support the findings of this study are available in the supplementary material of this article.
