# Supplementary material for: Manipulating Li2S Redox Kinetics and Lithium Dendrites by Core–Shell Catalysts under High Sulfur Loading and Lean‐Electrolyte Conditions
Source: Adv Sci (Weinh). 2023 Mar 18;10(14):2207442. doi: 10.1002/advs.202207442 (PMC10190580; doi:10.1002/advs.202207442)
Supplement: Supplementary file 1 — Supporting Information [file ADVS-10-2207442-s001.pdf]

ADVANCED SCIENCE
Open Access

Supporting Information

for *Adv. Sci.*, DOI 10.1002/advs.202207442

Manipulating Li_2S Redox Kinetics and Lithium Dendrites by Core–Shell Catalysts under High Sulfur Loading and Lean-Electrolyte Conditions

*Mengmeng Zhen, Kaifeng Li and Mingyang Liu**

Supporting Information

Manipulating Li_2S Redox Kinetics and Lithium Dendrites by Core-Shell Catalysts under High Sulfur Loading and Lean-Electrolyte Conditions

*Mengmeng Zhen, Kaifeng Li and Mingyang Liu**

Material Characterizations

The morphologies of the obtained samples were characterized by a scanning electron microscope (SEM, XL30 ESEM-FEG, USA) at 10.0 kV and transmission electron microscope (TEM, ARM200F, Japan). The thermogravimetric analysis (TGA, STA2500, NETZSCH, German) was performed to evaluate the thermal stabilities of the samples and the sulfur content of the cathode in an N_2 atmosphere from 30 °C to 700 °C. The N_2 adsorption-desorption data was measured at 77 K using a 30 Micromeritics ASAP 2020 C to determine the specific surface area, pore volume and pore size distribution. X-ray photoelectron spectroscopy was performed by PHI 5000 Versa Probe III with a monochromatic Al Kα X-ray source, with a base pressure better than 5×10^{-7} Pa for analysis. X-ray powder diffraction (XRD, PANalytical empyrean series 2, Netherlands) patterns were collected with CuKα radiation and Raman spectroscopy (LabRAM HR Evolution, France) were collected with 473 nm laser source. UV-vis spectroscopy of the solutions was collected by an ultraviolet and visible spectrophotometer (UV-3600). Elemental analysis was performed on the Thermo Fisher Scientific iCAP RQ inductively coupled plasma mass spectrometer (ICP-MS).

Visualized Adsorption of LiPSs

5.0 mM Li_2S_6 solution was obtained by dissolving Li_2S and S (molar ratio of 1:5) into 1,2-dimethoxyethane (DME) and 1,3-dioxolane (DOL) (volume ratio of 1:1) under vigorous stirring at 80°C for 24 h. 20 mg as-prepared interlayer materials were added into 5.0 mL Li_2S_6 solution.

Nucleation and Dissolution of Li_2S

0.5 M Li_2S_8 solution was obtained by dissolving Li_2S and S (molar ratio of 1:7) in tetraethylene glycol solvent under vigorous stirring at 60°C for 48 h. As-prepared samples, Super P and polyvinylidene fluoride (PVDF) (weight ratio of 8:1:1) were dispersed in NMP and coating on the Al foil with a diameter of 12 mm and dried at 60 °C for 12 h to be used as cathode. 25 μL LiTFSI electrolyte was added into the Li side and 25 μL Li_2S_8 solution was added into the cathode side.[S1] The cell was discharged galvanostatically to 2.06 V at 0.112 mA, and then discharged potentiostatically at 2.05 V until current decreased to 10^{-5} A. To investigate the dissolution of Li_2S, fresh cells were first discharged at a current of 0.10 mA to 1.80 V, and subsequently discharged at 0.01 mA to 1.80 V until full conversion of LiPSs into solid Li_2S. Then, the cells were potentiostatically charged at 2.40 V for the dissolution of Li_2S into LiPSs until charge current was below 10^{-5} A. [S2]

Preparation of CoNC@Co_9S_8NC modified separator

The as-prepared CoNC@Co_9S_8NC and PVDF with a weight ratio of 9:1 was mixed in NMP to form a homogeneous slurry and then coated onto polypropylene (PP) separators (Celgard 2400). The obtained CoNC@Co_9S_8NC modified separator was dried under vacuum at 60 °C for 12 h and cut into 16 mm circular disks (mass loading ≈ 1.85 mg cm^{-2}). CoNC and Co_9S_8NC modified separators were prepared through similar procedures.

Preparation of S cathode

CMK-3 and S with a weight ratio of 3:7 was added in 20 mL CS_2 solutions and then sonicated until the CS_2 solution evaporates completely. The mixture was heated 160 °C in an oven for 24 h under Ar atmosphere to get CMK-3/S. The composites, Super P and PVDF were mixed (with a weight ratio of 8:1:1) in NMP to form a slurry. Then, the slurry was coated on carbon cloth and then dried in an oven at 60 °C for 12 h. The mass loading of S was 3.2 mg cm^{-2} ~ 8.9 mg cm^{-2}.

Assembly of Li_2S_6 Symmetric Cells

Symmetric cells were assembled with two same electrodes of CoNC@Co_9S_8NC, CoNC and Co_9S_8NC. 0.2 M Li_2S_6 solution (in DME/DOL) solution containing 1.0 M

LiTFSI and 0.1 M $LiNO_3$ was used as the electrolyte. Cyclic voltammetry (CV) tests were carried out at scan rates of 0.5 mV s^{-1} and 20 mV s^{-1} between -1.0 V and 1.0 V on the CHI660E electrochemical workstation.

Electrochemical Tests

The electrochemical tests were carried out by using CR2032-type coin cells which were assembled in an Ar-filled glove box. The Li metal, as-prepared modified separator, and S cathode were used as anode, separator and cathode, respectively. Electrolyte was the 1 M lithium bis(trifluoromethanesulfonyl) imide (LiTFSI) and 0.1 M $LiNO_3$ in DOL/DME (1:1 by volume) solvents. The electrolyte/sulfur (E/S) was kept at ~12 μL mg^{-1}, or 4.5 μL mg^{-1} per cell. CV curves were tested at scan rates from 0.1 mV s^{-1} to 0.5 mV s^{-1} between 1.7 V and 2.8 V, and electrochemical impedance spectroscopic (EIS) were performed at a frequency range of 0.01 Hz-10 kHz on the CHI660E electrochemical workstation. The galvanostatic charge-discharge profiles were obtained using a Land battery tester with a voltage from 1.7 to 2.8 V *vs* Li^+/Li.

Computational Methods

The present first principle DFT calculations are performed by Vienna Ab initio Simulation Package (VASP) [S3] with the projector augmented wave (PAW) method. [S4] The exchange-functional is treated using the generalized gradient approximation (GGA) of Perdew-Burke-Ernzerhof (PBE) functional. The energy cutoff for the plane wave basis expansion was set to 450 eV and the force on each atom less than 0.02 eV/Å was set for convergence criterion of geometry relaxation.15 Å vacuum was added along the z direction in order to avoid the interaction between periodic structures. The Brillouin zone integration are performed using 3×3×1 and 3×2×1 k-point sampling for Co (111) and Co_9S_8 (440), respectively. The self-consistent calculations apply a convergence energy threshold of 10^{-5} eV. The DFT-D3 method was employed to consider the van der Waals interaction. [S5] Transition state searching were calculated using the climbing-image nudged elastic band (CI-NEB) method.

The adsorption energy of DME, DOL molecules and Li atom were calculated according to

$$\mathrm{E}_{ads} = E_{total} - E_{sub} - E_{mol}$$

where E_{total} is the total energy of the DME/DOL/Li adsorbed systems, E_{sub} and E_{Li} are the energies of the substrate and the isolated DME/DOL/Li, respectively.

Supplementary Figures and Tables

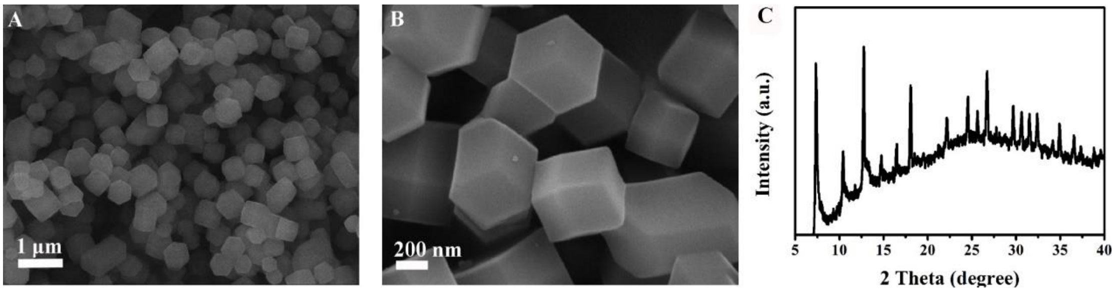

Figure S1. SEM images and XRD pattern of ZIF-67.

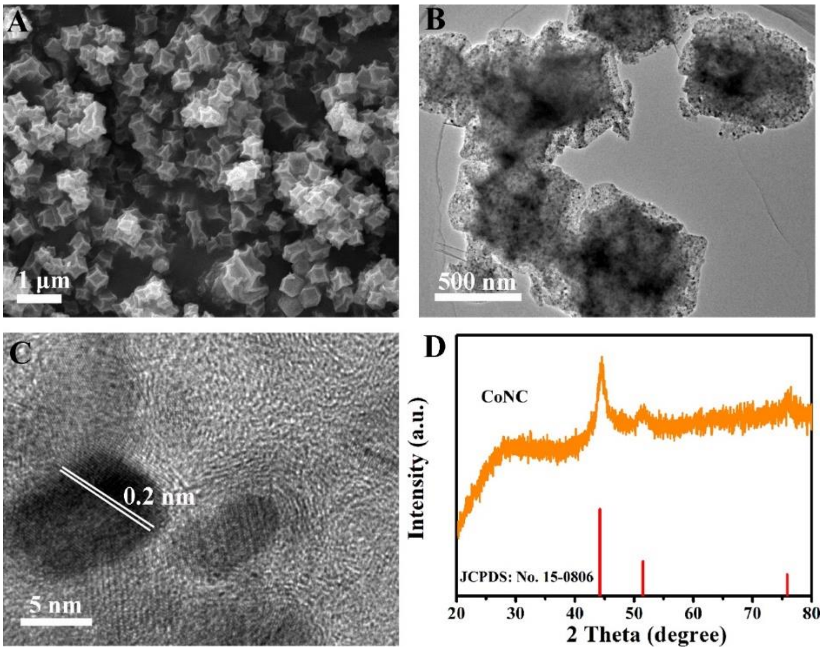

Figure S2. (A) SEM, (B~C) TEM images and (D) XRD pattern of CoNC.

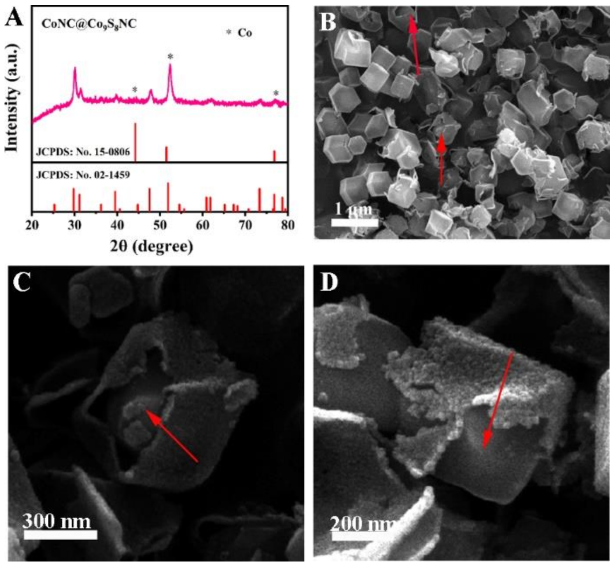

Figure S3. XRD pattern (A) and SEM images (B~C) of CoNC@Co_9S_8NC.

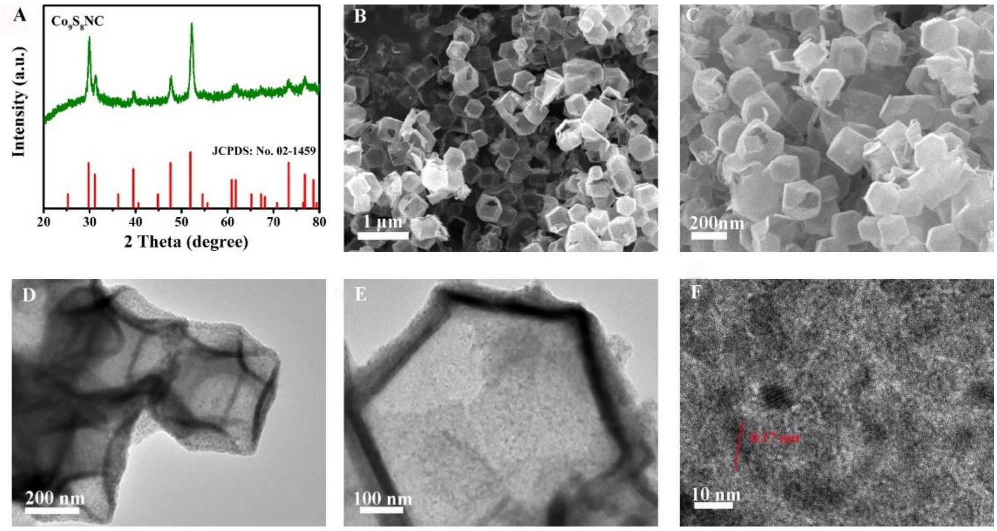

Figure S4. XRD pattern, SEM images and TEM images of Co_9S_8NC.

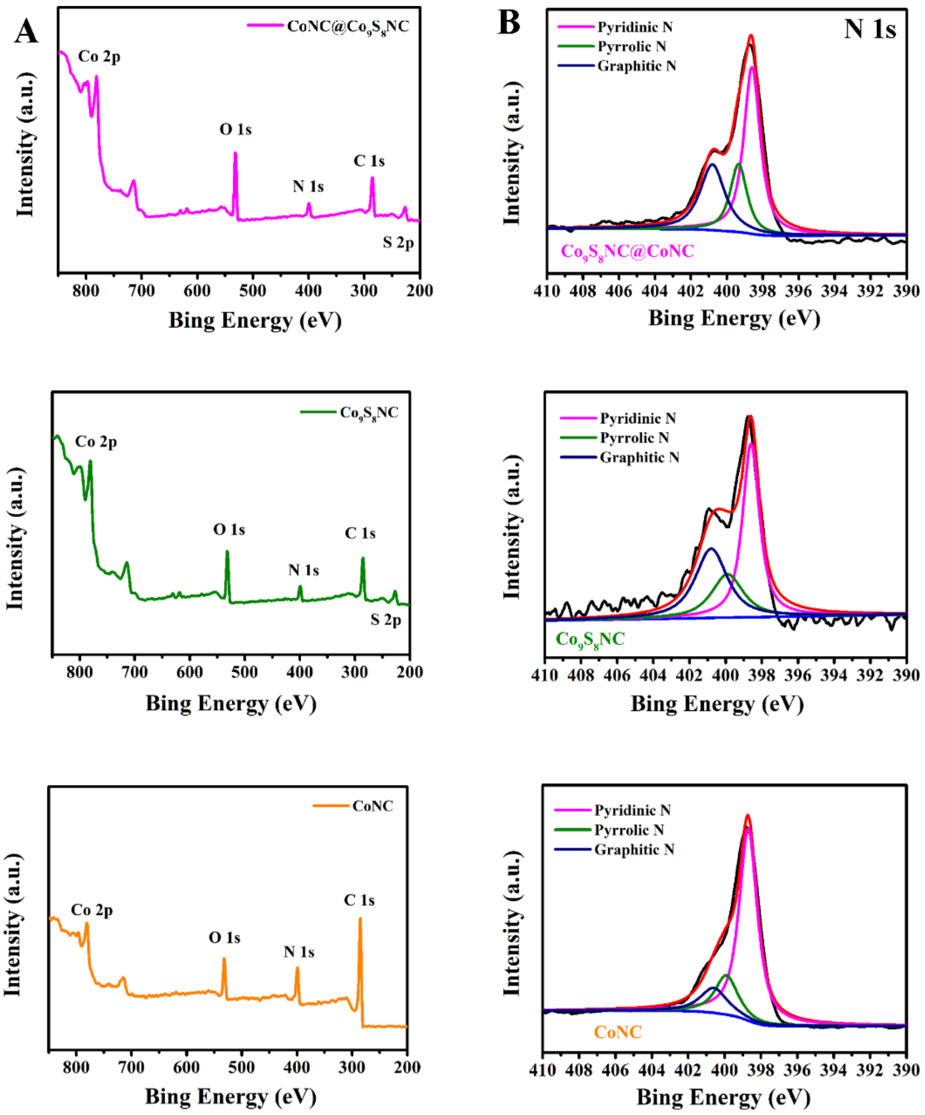

Figure S5. XPS spectrum and high-resolution N 1s XPS spectra of different samples.

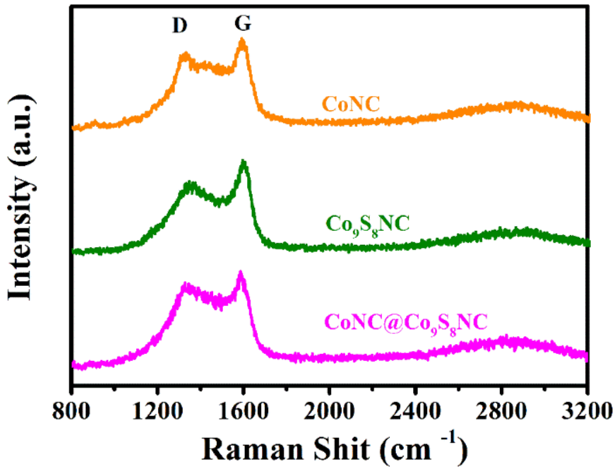

Figure S6. Raman spectra of different samples.

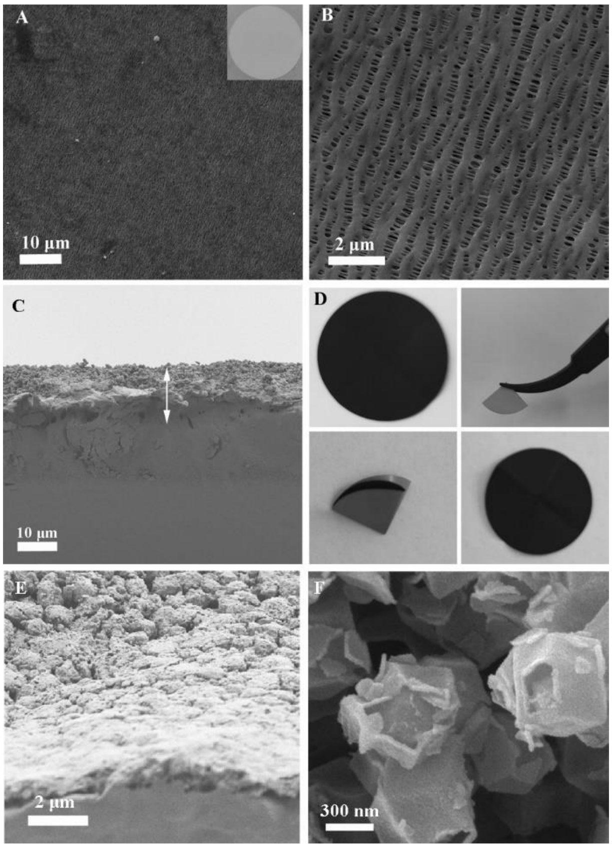

Figure S7. (A and B) SEM images of the surface of blank separator, (C and D) the cross section and the bending of CoNC@Co_9S_8NC modified separator, and (E and F) SEM images of the surface of CoNC@Co_9S_8NC modified separator.

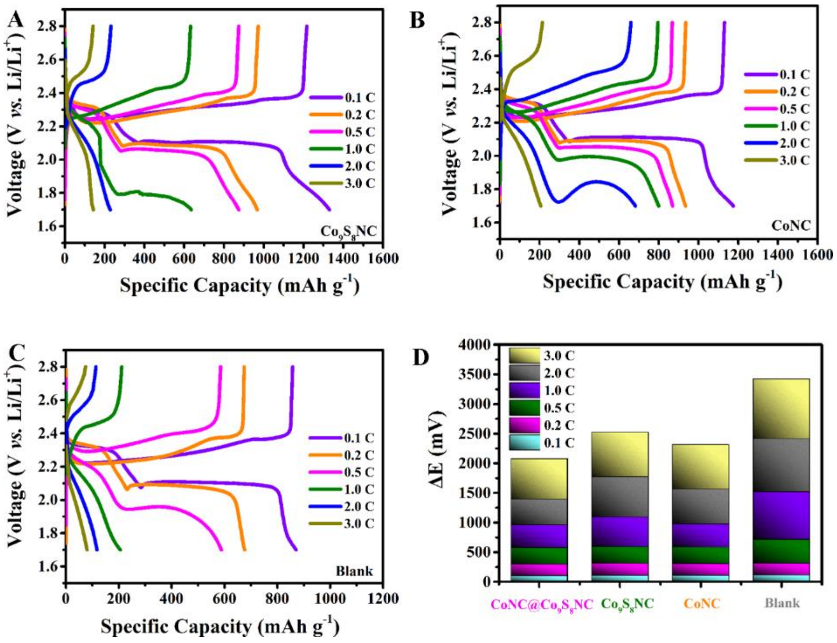

Figure S8. (A~B) Charge/discharge plateaus and ΔE of cells with different modified separator

and (D) current rates from 0.1 C to 3.0 C.

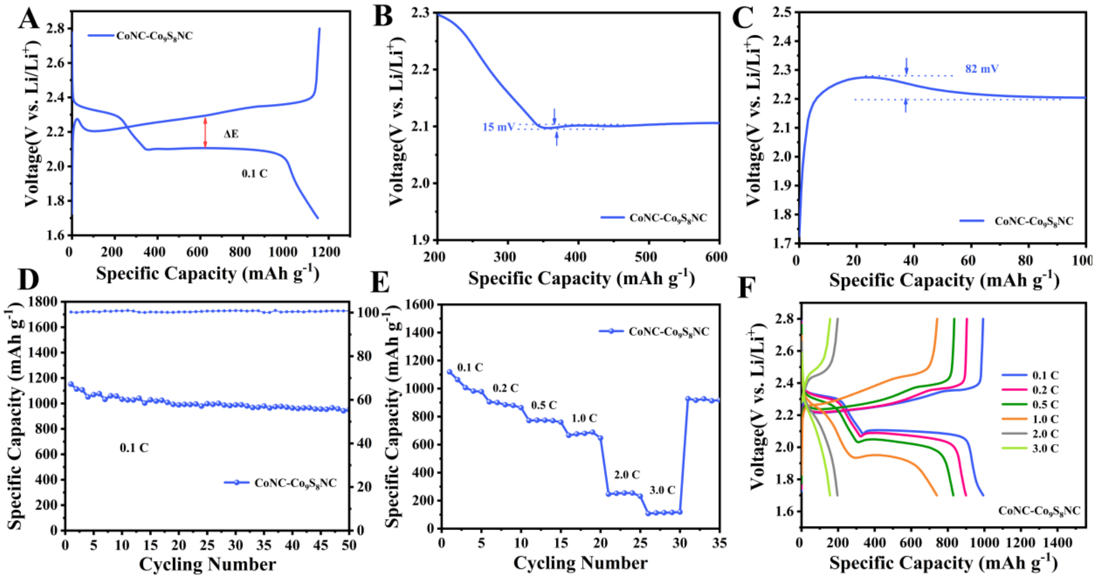

Figure S9. (A~C) charge/discharge curves, (D) cycling performances, (E) rate capabilities and (F) charge/discharge curves of the cell with CoNC-Co_9S_8NC modified separator at different current rates.

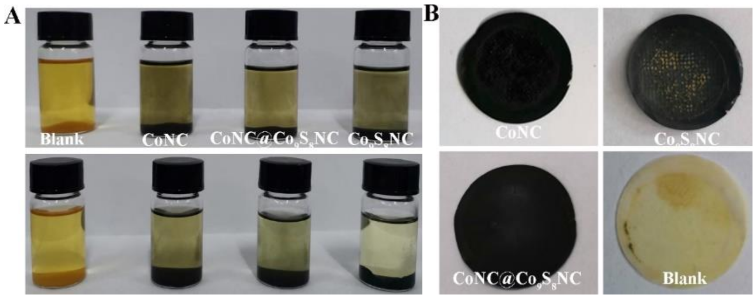

Figure S10. (A) The digital photos of Li_2S_6 solution treated with different interlayer materials and (B) the surfaces of different modified separator after cycling.

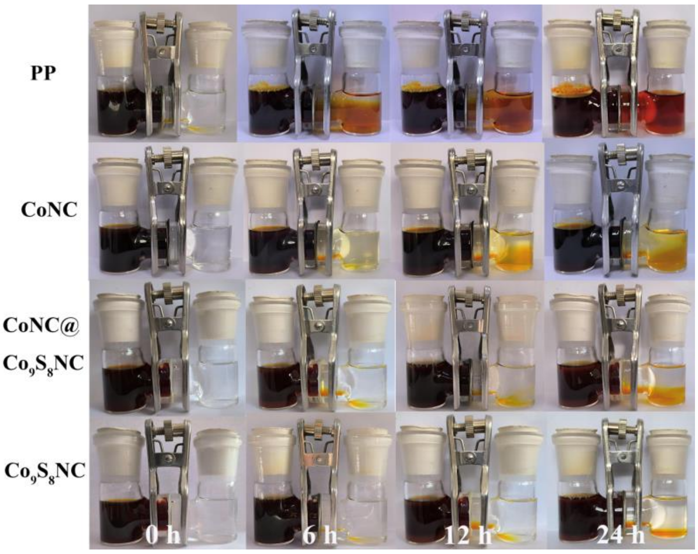

Figure S11. Optical pictures of Li_2S_6 permeation experiments on different separators.

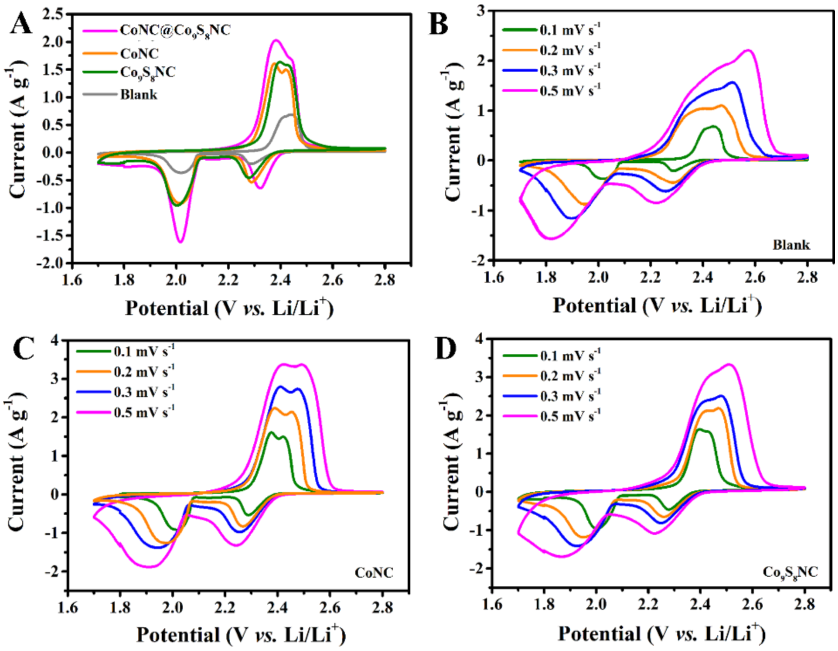

Figure S12. (A) CV curves of the cells with different separators at a scan rate of 0.1 mV s^{-1}, (B~D) CV curves of the cells with different separators at different scan rates from 0.1 to 0.5 mV s^{-1}.

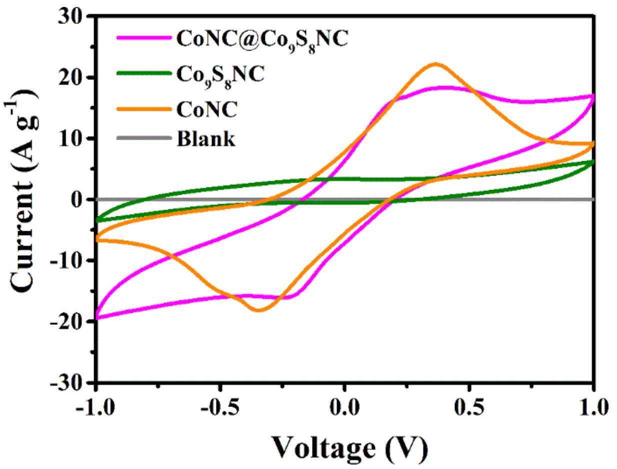

Figure S13. CV curves of Symmetrical cells with different electrodes at a scan rate of 20 mV s^{-1}.

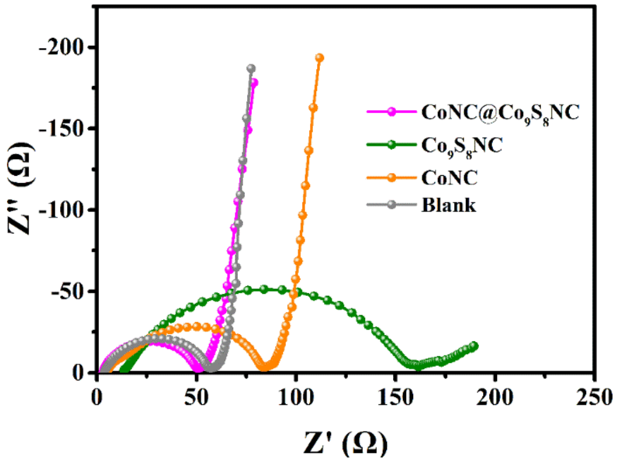

Figure S14. EIS curves of the cells with different separators.

References

[S1] W. Wang, L. Y. Huai, S. Y. Wu, J. W. Shan, J. L. Zhu, Z. G. Liu, L. G. Yue, Y. Li, *ACS Nano* **2021**, *15*, 11619.

[S2] C. Q. Zhang, J. J. Biendicho, T. Zhang, R. F. Du, J. S. Li, X. H. Yang, J. Arbiol, Y. T. Zhou, J. R. Morante, A. Cabot, *Adv. Funct. Mater.* **2019**, *29*, 1903842.

[S3] G. Kresse, *J. Non-Cryst. Solids* **1996**, *207*, 833.

[S4] K. Seifert, J. Hafner, G. Kresse, *J. Non-Cryst. Solids* **1996**, *207*, 871.

[S5] S. Grimme, J. Antony, S. Ehrlich, H. Krieg, *J. Chem. Phys.* **2010**, *132*, 154104.
